# Soil humic acids degrade CWD prions and reduce infectivity

**DOI:** 10.1371/journal.ppat.1007414

**Published:** 2018-11-29

**Authors:** Alsu Kuznetsova, Catherine Cullingham, Debbie McKenzie, Judd M. Aiken

**Affiliations:** 1 Agricultural, Life and Environmental Sciences Faculty, University of Alberta, Edmonton, AB, Canada; 2 Faculty of Science, University of Alberta, Edmonton, AB, Canada; Creighton University, UNITED STATES

## Abstract

Chronic wasting disease (CWD), an environmentally transmissible, fatal prion disease is endemic in North America, present in South Korea and has recently been confirmed in northern Europe. The expanding geographic range of this contagious disease of free-ranging deer, moose, elk and reindeer has resulted in increasing levels of prion infectivity in the environment. Soils are involved in CWD horizontal transmission, acting as an environmental reservoir, and soil mineral and organic compounds have the ability to bind prions. Upper horizons of soils are usually enriched with soil organic matter (SOM), however, the role of SOM in prion conservation and mobility remains unclear. In this study, we show that incubation of PrP^CWD^ with humic acids (HA), a major SOM compound, affects both the molecular weight and recovery of PrP^CWD^. Detection of PrP^CWD^ is reduced as HA concentration increases. Native HA extracted from pristine soils also reduces or entirely eliminates PrP^CWD^ signal. Incubation of CWD prions with HA significantly increased incubation periods in tgElk mice demonstrating that HA can reduce CWD infectivity.

## Author summary

Chronic wasting disease (CWD) is a contagious prion disease affecting several species of captive and wild cervids. Environmental prion contamination plays a major role in increasing incidence of CWD, with CWD infectivity being released into the environment by decaying carcasses, or shedding of biological fluids including urine, feces, and saliva. Horizontal transmission of CWD involves soils as an environmental reservoir of infectivity. Here, we tested the role of a soil organic matter compound, humic acid, for its ability to bind CWD prions and impact infectivity. A wide range of humic acid concentrations were examined representing the extensive spectrum of humic acid levels present in native soils. We found that incubation of CWD prions with high concentrations of humic acids (>2.5 g L^-1^) decreases the both CWD-prion signal and infectivity, whereas lower levels of humic acids did not significantly impact protein stability or infectivity. Our study provides new insights into soil-prion interactions, prions persistence in soil, and their bioavailability to grazing animals.

## Introduction

Chronic wasting disease (CWD), a fatal prion disease affecting free ranging white-tailed deer, mule deer, elk, moose and reindeer as well as farmed cervids, is the only spongiform encephalopathy (along with scrapie) that is environmentally transmitted. Currently, CWD-infected cervids are present in 3 provinces of Canada, 25 US states, South Korea, Norway and Finland, and its geographic range continues to expand among free-ranging cervids. Evidence suggests that horizontal transmission of CWD involves soils as an environmental reservoir of infectivity [1–4]. Soil-related CWD transmission in cervids may occur orally [5], although intranasal and aerosol routes of transmission are also possible [6,7]. The CWD agent may enter the soil via alimentary secretions, blood, and decomposing infected carcases [8]. While prion infectivity persists in the soil for a few years in laboratory conditions [9], infectivity of soils in CWD-endemic regions remains largely unknown. Different soil compounds, mineral and organic, can differentially bind prions and change their infective properties [1]. Montmorillonite (mte) mineral particles bind prions avidly and increase their infectivity [10]; kaolinite (kte) and quartz microparticles (common soil minerals) also may increase disease transmission [11]. Upper horizons of soils are usually enriched with soil organic matter (SOM) but the role of SOM in prion conservation and mobility remains unclear. SOM is defined as all biologically derived organic matter that resides within the soil matrix and is divided into living and non-living components [12]. Humus is the most important and specific part of SOM; it is a mixture of amorphous organic materials that contains identifiable biomolecules (e.g. polysaccharides, lipids, proteins etc.) and non-identifiable molecules (humic substance) [13]. Humus is an extraordinarily complex, molecularly flexible material that can be fractionated into specific humic substances: fulvic acids (FA), humic acids (HA), and humin. Humic acids are comprised of weak aliphatic (carbon chains) and aromatic (carbon rings) organic acids which are insoluble in water under acid conditions, but soluble in water under alkaline conditions. Humic acids have average molecular weights varying from 10kDa-103kDa for soil-derived material [14]. They are considered to be flexible linear polymers that exist as random coils with cross-linked bonds. In water solution, the HA are large, dynamic supramolecular associations, held together by hydrophobic interactions, which are easily disrupted and capable of exhibiting micellar properties [15].

Due to the wide variety of soil types, composition and amount of SOM differs between locations. For example, in western Canada, there are at least 12 great groups of soils [1] and each is comprised of different amounts and types of organic compounds. SOM content in the upper soil horizons vary from 0.5% in the surface horizon of Regosolic soils, to 30% in the LHF (plant litter) horizon of most soils in the northern part of Canada. The ratio of different humic compounds also varies across soil types. In soils where the vegetative (biologically productive) period is longer, the humus has more HA than FA (e.g. in Chernozems) compared to soils that are less biologically active (due to non-optimal temperatures and moisture regimes), where the humus contents are mostly FA (e.g. in Luvisols) [16,17]. For Chernozemic soils of Northern American CWD-endemic regions, HA abundance was estimated between 0.75%-1.4% [18]. In Luvisolic and Brunisolic soils, the upper horizon contains high amounts of SOM, but insignificant amounts of HA [19]; for boreal region soils, the HA content is estimated as 0.1–0.7% [16].

Since most of SOM is concentrated in the surface soil horizon and HA is a major component of SOM, it likely plays an important role in the fate of shed prions. Although the absolute concentration of HA in soil is significantly less than the abundance of mineral particles, HA is more biologically and chemically active and can adsorb on mineral particles creating films on the surfaces and masking them. Analyses of HA-prion interactions, and their impact on CWD infectivity, are important factors in determining the fate of PrP^CWD^ in soil environments. While a series of studies have investigated prion binding to soils varying in SOM content, the direct interaction of HA with PrP^CWD^ has not been studied [20,21]. Studies using non-infectious recombinant PrP (recPrP) indicated that HA and other SOM compounds have a strong affinity to the recPrP [22–26]. However, due to difference in structure of infectious and non-infectious prions, they may interact differently with SOM compounds. It has previously been shown that hamster prions, when incubated with low concentrations of HA, remain infectious to hamsters with little biological impact on the incubation period [27]. The objective of this study was to determine, by examining a more complete range of HA concentrations, how interactions with HA affects PrP^CWD^ and resulting infectivity.

## Results and discussion

### Soil organic material degrades CWD prions

**Fig 1:**
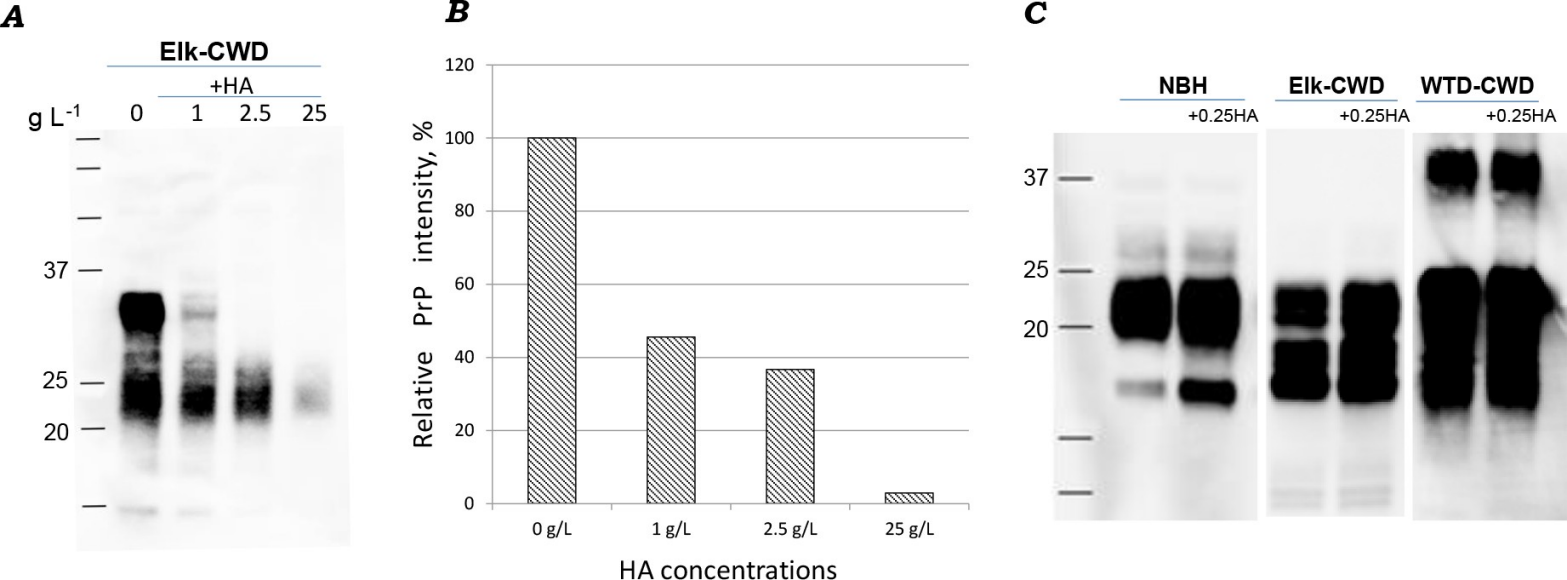
Interactions of PrP^CWD^ with humic acids (HA) affect PrP^CWD^ recovery and molecular weight. (A) PrP^CWD^ signal was reduced with increasing HA concentration. (B) Quantification of PrP signal (using ImageJ software). (C) Low concentration of pure HA (0.25 g L^-1^) does not affect PrP recovery and molecular weight. Identical amounts of 10% brain homogenate (BH^CWD^ and uninfected control (NBH)) were incubated with water (control) and HA (0.25 g L^-1^ or 1 g L^-1^, 2.5 g L^-1^ and 25 g L^-1^) overnight at 4°C. Samples were analyzed by western blot with Bar224 antibody.

The interactions between prions and HA were first analyzed using commercially available pure HA. HA levels used in this study mimicked levels naturally present in soils (1g L^-1^-25g L^-1^). Humic acids and brain homogenates (CWD infected and uninfected controls) were incubated overnight. PrP^CWD^ signal decreased with increasing HA concentration with the ~35 kDa molecular weight band (diglycosylated PrP) not visible at the highest concentration (25 g L^-1^) of HA (Fig 1). The quantity of PrP signal for Fig 1A declined to 33% following incubation with 2.5 g L^-1^ HA, and for 25 g L^-1^ HA the decline was to 5% (Fig 1B).

**Fig 2:**
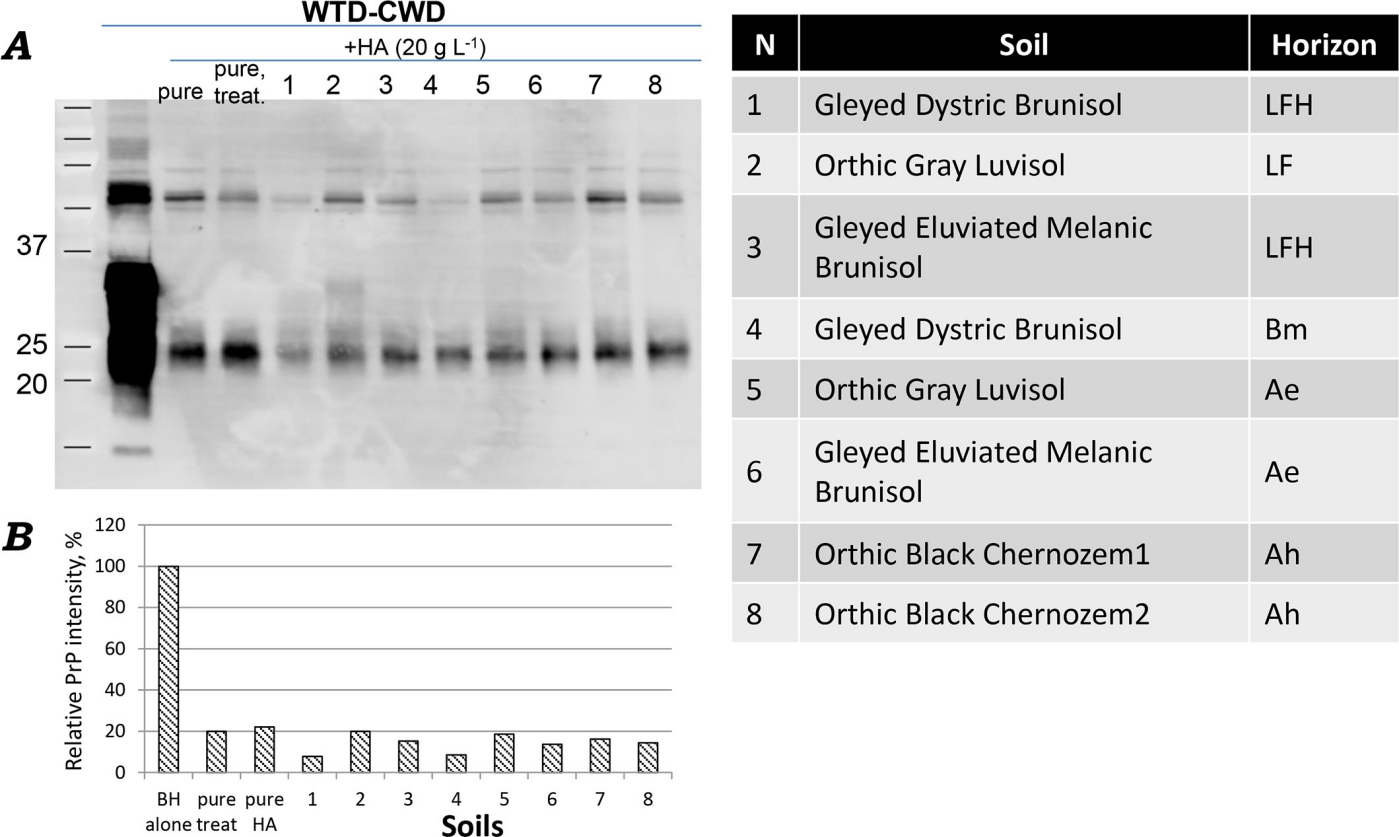
(A) HA, extracted from different soils and normalized to 20 g L^-1^, reduces PrP^CWD^ signal. (B) Quantification of PrP signal (using ImageJ software). Identical amounts of 10% brain homogenate (BH^CWD^) were incubated overnight at room temperature with water (control), pure HA and HA extracted from Alberta soils: 1 –mountain Brunisol, horizon LF; 2 -northern Luvisol, LFH; 3 –northern Brunisol, LFH; 4—mountain Brunisol, Bm; 5 –northern Luvisol1, Ae; 6 –northern Brunisol, Ae; 7 –central Chernozem1, Ah; 8 –south Chernozem2, Ah. Pure HA was included as a control, (20 g L^-1^) from two preparations. One solution (first lane, “pure HA”) was prepared directly from the HA powder, and the second (second lane, “pure treat”) solution was treated similar to the HA extraction procedure (Materials and Methods). Samples were analyzed by immunoblotting with Bar224 antibody.

Due to the variability of HA amount and composition in soils, the effects of native soil HA on PrP^CWD^ might vary. Native HA were extracted from different soils from boreal, prairie and mountainous regions of western Canada (S1 Table). The amount of extracted HA varied amongst the soils. As expected, HA extracted from surface humic Ah horizon of Chernozemic soils had maximal concentrations of HA, 19–22 g L^-1^, and the lowest concentrations of HA were in the eluvial horizon Ae of Luvisol from the boreal ecozone, 0.2 g L^-1^. All soil-extracted HA decreased PrP^CWD^ signal but to differing levels (S2 Fig). Stronger signal was observed with lower concentrations of HA (e.g. in the LF horizons of Luvisol and Brunisol), while the weakest signals were observed after incubation with higher concentrations of HA, (similar to the results with commercially available pure HA). LF horizons have the highest amount of total organic carbon (34–38%) but are a negligible source of HA because they are composed primarily of degraded materials; the non-decomposed organic fraction has minor chemical importance because its intact structure exhibits a relatively small surface area [28]. To determine if HA from different soils has similar effects on reducing PrP^CWD^ signal, we normalized the HA concentration to 20 g L^-1^. After incubation with normalized soil HA, PrP^CWD^ signal was also reduced (Fig 2), and differences between HA in different soils was not observed. Pure HA included as a control at the same concentration (20 g L^-1^) showed a similar effect on PrP signal. In all cases, the ~30kDa PrP band was degraded, and 22–23 kDa protein remained.

There are several potential explanations for the observed decline in PrP^CWD^ signal upon incubation with humic acids. One possibility is that a component of SOM interferes with PrP^CWD^ during immunoblotting which could alter migration, limit entry into the gel, or interfere with membrane transfer [27]. We found that PrP^CWD^ migrated as expected (20–35 kDa) with or without HA, thus altered migration during SDS-PAGE is not the reason for decreasing PrP signal. Staining the membranes with Coomassie Blue showed that the majority of proteins entered into the gel and did not remain in the wells (S3 Fig), indicating there is no effect of entry into the gel. The observed loss of PrP signal with increasing HA concentration could also result from the encapsulation of PrP^CWD^ impacting prion migration, alternatively HA may cause partial degradation of PrP^CWD^ so that only a portion remains detectable. The exact mechanisms of these processes are still unclear [3], but there is evidence to suggest that either is possible. Tomaszewski et al. [29] proposed negatively-charged HA could encapsulate positively-charged proteins and preserve their activity. Another study [25] has shown HA-like substances copolymerize with recPrP and irreversibly incorporate it into their structure, creating complexes which decrease the efficiency of recPrP recovery. The PrP insolubilization and co-precipitation by aggregation with HA without altering PrP secondary structure, which potentially can decrease prion detectability and reduce their bioavailability, also were discussed [26,27]. Similar prion interactions with another high-organic matrix (e.g. compost) revealed PrP^CWD^ degradation following 230-days of composting [30].

We also assessed the effects of high concentration HA on PrP^res^ (S4A Fig). The results showed that HA degraded PrP^res^ with mono- and unglycosylated forms degraded more rapidly. A similar effect of HA was found for deglycosylated PrP^CWD^ (S4B Fig). Incubation with 25 g L^-1^ HA decreased the intensity of the higher molecular band (25kDa) but also affected the lower weight bands as shown by a decline in intensity of the 13-16kDa bands.

### HA can degrade CWD infectivity

**Fig 3:**
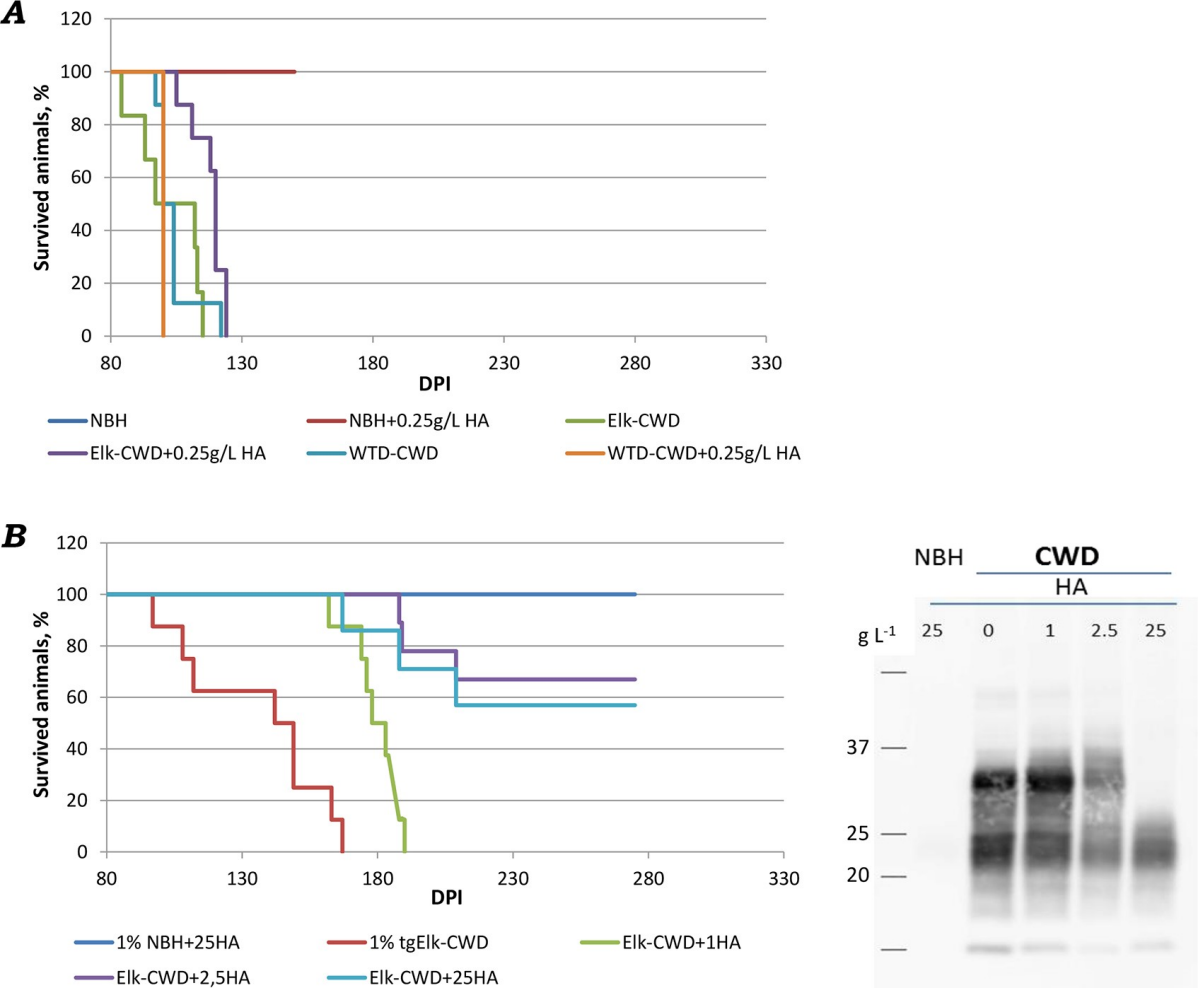
Infectivity bioassays. (A) Intracerebral inoculation into tgElk mice. Mice inoculated with 0.16% Elk-PrP^CWD^ alone exhibited an incubation time of 102±13 dpi; the same amount of PrP^CWD^ with HA presented with clinical signs at 118±7 dpi; WTD-CWD inoculum resulted in incubation period 104±8 dpi while mice inoculated with WTD-CWD+HA were CWD-positive at 100 dpi. Mice inoculated with NBH with and without HA remained healthy throughout the course of the experiment. (B) Intraperitoneal inoculation into tgElk mice revealed significant differences between mice inoculated with untreated BH and BH pre-incubated with lowest (1 g L^-1^) concentration of HA. Half of the mice inoculated with a higher HA concentration showed clinical signs and were euthanized; another half did not show clinical signs. Immunoblot shows inoculum prepared for inoculation: BH (10% Elk-CWD) without and with 1g L^-1^, 2.5 g L^-1^ and 25 g L^-1^ concentrations of HA were incubated overnight.

Our initial bioassays challenged animals (tgElk mice) by the intracerebral route. Due to brain toxicity of HA, only the relatively low concentration (0.25 g L^-1^) was examined. Two CWD strains were studied, elk prions (source: infected elk brain) and white-tailed deer (WTD) prions (source: tg mice). Control mice were incubated with uninfected NBH and NBH+0.25HA; they did not show clinical signs and were euthanized at the end of the experiment (at 175dpi). Mice inoculated with 0.16% BH from CWD infected elk exhibited clinical symptoms earlier than tg-mice inoculated with 0.16% CWD infected elk BH + 0.25 g L^-1^ HA. The difference between treatments was 16 days (102±13 dpi vs 118±7 dpi) suggesting a slight reduction in effective titer, but due to the overlap in incubation periods, the difference was not statistically significant. For WTD-CWD inoculum, the incubation periods were similar: 104±8 (dpi) without HA and 100±0 dpi with HA (Fig 3A). This is supported by the results from immunoblotting where there is no reduction in PrP^CWD^ intensity for these inoculums. Similar to the study of hamster prions/humic acids [27], we identified a slight, biologically insignificant (<1 log) change in infectivity.

To examine the impact of higher HA concentrations on CWD infectivity, mice were infected by intraperitoneal route. Elk prions were incubated with 1 g L^-1^ HA, 2.5 g L^-1^ HA and 25 g L^-1^ HA. Control reactions included uninfected brain homogenate as well as CWD-elk prions incubated without HA. Western blot analysis confirmed a loss of signal with increasing HA concentration (Fig 3B). This result is similar to what we observed previously, where the strongest PrP signal was detected for the sample without humic acids, and the signal decreased with increasing HA concentration. Animal bioassay of the samples showed a concomitant decline in CWD infectivity with increasing concentration of HA. Significant differences (*p*<0.001) between mice inoculated with CWD brain homogenate control (no HA) and infected brain homogenate treated with 1g L^-1^ HA (Fig 3B); the incubation period for BH (control) was 154±10 days post inoculation (dpi), while for BH pre-incubated with 1 g L^-1^ HA it was 180±10 dpi. Only half of the mice inoculated with BH pretreated with higher HA concentrations (2.5 g L^-1^ and 25 g L^-1^) exhibited clinical signs within the 280-days (starting at 167–188 dpi). We analyzed brains harvested from these mice and all were positive for PrP^res^ (after PK digestion) by western blot. The remaining mice (5 out of 10 for 2.5 g L^-1^ and 5 out of 8 for 25 g L^-1^) were euthanized at 280 dpi without clinical signs. PrP^res^ was not identified in their brains. Control animals inoculated with normal (uninfected) BH (NBH) pre-incubated with 25 g L^-1^ HA survived during the course of the bioassay (>280 dpi). This bioassay clearly demonstrates a decline in CWD infectivity after incubation with higher concentrations of HA.

Due to the complexity of soil, it is impossible to distinguish the influence of each compound separately because of their overlapping influence and sometimes different impact on prions. In soil surface horizons, mineral clay particles are usually covered with films of soil organic matter (SOM) (or iron/manganese oxides, or carbonates depending on soil type). Thus detection of HA degrading ability on PrP is an important first step to understanding soil-prion interactions. Similar to previous studies [2,4,10,31,32] where prion interactions with pure minerals were investigated, our research will help to understand the complexity of soil-prion interactions. It is important to analyze how HA changes binding capacity of minerals and look in this direction for our future experiments. The adsorption of organic constituents by clay particles leads to the formation of organo-mineral complexes; they are abundant and common in soils, and adsorption of humic substances by clays has been extensively investigated [33,34]. Organic substances can bind to clays through a variety of mechanisms that depend on the properties of the organic compounds and the mineral surface. These interactions can change the adsorption capacity and the reactivity of minerals [35]. The surface chemistry of organo-mineral complexes is dominated by adsorbed organic matter that masks the properties of the supporting minerals to varying extents [36,37]. Therefore, the fate of prions in soil (binding and mobility) may be regulated primarily by interactions with organo-mineral complexes, and the effect of prion binding by organo-mineral complexes and its persistence in the soils needs additional study to determine its implications on the bioavailability of PrP^CWD^.

This study shows that a common soil component, HA, altered the mobility and abundance of PrP^CWD^ with a concomitant decline in CWD infectivity. A wide range of HA concentrations were tested, from 0.25 to 25 g L^-1^, representing the spectrum of HA present in native soils. The lowest HA concentrations (<1 g L^-1^) occur in boreal and tundra soils while high HA concentrations (>20 g L^-1^) are present in soils of prairie grassland. We have shown that high concentrations of HA (>2.5 g L^-1^) decrease both PrP^CWD^ signal and prion infectivity. HA extracted from a variety of pristine soils also reduced PrP^CWD^ signal, suggesting that a similar mechanism of prion-HA interaction can occur in different types of soils.

The lack of detailed knowledge on the composition and structure of HA makes it difficult to identify the specific relationships between the structure and activity of these substances. Although we showed that native HA purified from different soils have a similar effect on prions, the soil diversity and complexity with varying mineral and organic compounds may affect the HA degradation ability, and enhance PrP^CWD^ persistence in soil, and also may contribute to the migration of prions in the soil profile that could change prion bioavailability to grazing animals. This study further emphasizes the complexity of soil-prion interactions, where soil minerals bind prions and enhance infectivity while the organic compounds can degrade CWD-prions.

## Materials and methods

### HA sources and extraction

For HA-prion incubation experiments, we used commercially available HA (Sigma-Aldrich, cat. #53680; referred to as pure HA), dissolved in deionized water at the following concentrations: 1 g L^-1^, 2.5 g L^-1^, and 25 g L^-1^. These concentrations were chosen to reflect actual concentrations of HA in soils (1–2.5 g L^-1^ or less, northern boreal and tundra regions; 25 g L^-1^, prairie Chernozemic soils).

HA were also extracted from the surface horizons of 6 pristine soils: two Chernozems from the prairie region, a Luvisol and Brunisol from the boreal region, and a Brunisol from the mountainous region. Bulk soil samples from two upper horizons were collected at 6 sites in Alberta, Canada (S1 Fig). Each site had native vegetation and soil profiles that were undisturbed. Soil samples were air-dried and sieved to collect material <2 mm. Selected soil characteristics are provided in S1 Table. Soil texture was determined by gravimetric method with hydrometer; mineral composition of clay fractions was detected by X-ray diffraction (XRD) analyses in EAS (University of Alberta) using the Rigaku Geigerflex powder diffractometer. The extraction followed the protocol recommended by the International Society of Humic Substances [38]. Briefly, soil was pre-incubated in 1N HCl followed by a multistep extraction procedure: (i) extraction with 0.1N NaOH at room temperature overnight; (ii) centrifugation to collect the supernatant; (iii) acidification of the supernatant with 2M HCl; (iv) precipitation of HA overnight; and (v) separation of HA (precipitate) from FA (supernatant) by centrifugation. The NaOH extraction followed by acidic separation was repeated until the solution was clear (2–3 more times) to assure that all humic and fulvic acids were extracted. All centrifugation was carried out at 5000*g* for 10 minutes. Obtained HA pellets were combined and resuspended in water, resulting in varying concentrations of HA between collected soils. For incubation experiments with normalized HA concentrations, the HA pellets were weighed and re-suspended in water to adjust the concentration to 20 g L^-1^.

### Prion preparation, incubation and immunoblotting

CWD agent was obtained from infected brain tissues of elk [39], transgenic tgElk mice (expressing 132MM elk PrP, #2045 from already-existing collection in CPPFD) or infected transgenic tg33 mice (expressing wild type white-tailed deer PrP, #1268 from previous existing collection in CPPFD). Uninfected controls were brains of uninfected transgenic tg33 and tgElk mice. Brain tissues were homogenized (10% w/v) in water, and then were clarified at 800*g* for 5 min before experiments were initiated [4]. Identical amounts of 10% brain homogenate (BH^CWD^, or uninfected: NBH) were incubated with water (control) and HA (1g L^-1^, 2.5 g L^-1^ and 25 g L^-1^) at 4°C. For all experiments (unless otherwise specified), the BH and HA were incubated 24 hours. Following incubation, samples were analyzed by western blot: samples (10 μL) were resolved on 12-well 12% NuPAGE bis-Tris gels (Invitrogen), transferred to PVDF membrane and probed with anti-PrP antibody Bar 224 (diluted 1:20 000; Bertin Pharma). Quantitative analyses of western blot images were performed using ImageJ software (https://imagej.nih.gov/ij/index.html), which output the net intensity and area of each blot. Net intensities of the samples were normalized as a percent of the untreated controls run on the same gel.

### PNGase and Proteinase K treatment

Proteinase-resistant PrP^CWD^ was identified by digestion of 10 μL of brain homogenate with 3.5 μg of Proteinase K (PK) (Roche) for 45 minutes at 37°C in a volume of 50 μL (50 mg/ml PK final concentration). Digestion was terminated by addition of 10 μl of AESBF (proteinase inhibitor, #A8456 Sigma-Aldrich). Deglycosylation of PrP^CWD^ was performed with PNGase F kit (#P0704S, BioLabs Inc.) following manufacturer’s protocol (https://international.neb.com/protocols/2014/07/31/pngase-f-protocol).

### Ethics statement

All work with animals was performed in compliance with the Canadian Council on Animal Care Guidelines and Policies. All procedures involving animals were reviewed and approved by the Health Sciences Animal Care and Use Committee of the University of Alberta under protocol “Etiology and Pathogenesis of Prion Diseases” AUP # 914.

### Infectivity bioassay

Infectivity of CWD incubated with HA was determined by intraperitoneal or intracerebral infection of tgElk mice. To test low concentration of HA, 44 tgElk mice were inoculated intracerebrally. The inocula (10% elk-CWD and WTD-CWD BH) were incubated with 2.5 g L^-1^ HA, then diluted 10-fold to reach a non-toxic concentration of HA, pasteurized 10 min at 80°C, and 25 μL used to intracerebral inoculate tgElk mice. Intraperitoneal route was used to test higher concentrations of HA. For this experiment, BH (10% elk-CWD) was incubated overnight with varying concentrations of HA (1g L^-1^, 2.5 g L^-1^ and 25 g L^-1^), pasteurized 10 min at 80°C, and 100 μL used to intraperitoneal inoculate tgElk mice. Equivalent amounts of BH^CWD^ or uninfected BH (incubated with 25 g L^-1^ HA) were used as inoculate control. In both bioassays, mice were monitored daily for the onset of clinical symptoms and euthanized upon confirmed clinical disease. Brains from clinically positive mice and uninfected controls were analyzed for protease-resistant PrP (PrP^res^) by immunoblotting as described above.

## Supporting information

S1 Table Properties of collected soil samples. (DOCX)

S1 Fig Location of soil collection sites in Alberta (Canada). (TIF)

S2 Fig (A) Interaction of PrP^CWD^ with pure HA and with HA extracted from different soils affects PrP^CWD^ recovery and molecular weight. (B) Soil types used for HA extraction. Identical amounts of 10% BH^CWD^ were incubated with water (control) and pure HA (25 g L^-1^ as a control) as well as HA extracted from the soils overnight at 4°C. Samples were analyzed by western blot with Bar224 antibody.
(TIF)

S3 Fig Staining with Coomassie Blue demonstrates that decreases in PrP^CWD^ signal were not due to interference with protein fractionation. (TIF)

S4 Fig (A) High concentration of HA affects PrP^res^ (after PK- digestion): mono- and unglycosylated forms degraded faster. (B) High concentration of HA affects deglycosylated PrP^CWD^ (after PNGase treatment).
(TIF)
