# Supplementary material for: Soil humic acids degrade CWD prions and reduce infectivity
Source: PLoS Pathog. 2018 Nov 29;14(11):e1007414. doi: 10.1371/journal.ppat.1007414 (PMC6264147; doi:10.1371/journal.ppat.1007414)
Supplement: S1 Table — (DOCX) [file ppat.1007414.s001.docx]

Table S1. Properties of collected soil samples

| **Soil** | **Location in Alberta, Canada** | **Horizon** | **pH** | **TOC, %** | **HA content, g L^-1^** | **Texture, clay content %** | **Mineral composition of clay fraction** |
| --- | --- | --- | --- | --- | --- | --- | --- |
| Gleyed Eluviated Melanic Brunisol | Boreal region, Hangingstone river | LFH | 4.0 | 35 | 3.8 | N/A (org.hor.) | N/A (org.hor.) |
|  |  | Bf | 5.3 | 1.3 | 0.5 | Silt loam, 17% | Mica-illite |
| Orthic Grey Luvisol | Boreal region, Wapasu creek | LF | 6.3 | 34 | 3.1 | N/A (org.hor.) | N/A (org.hor.) |
|  |  | Ae | 3.5 | 0.8 | 1.5 | Silt loam, 28% | Mica-illite |
| Orthic Black Chernozem | Parkland region, Leduc | Ah | 7.3 | 4.0 | 19.0 | Silt loam, 21% | Montmorillonite - kaolinite |
| Orthic Black Chernozem | Prairie region, Kneehill county | Ah | 7.9 | 5.3 | 22.1 | Clay loamy-clay, 34% | Montmorillonite - kaolinite |
| Gleyed Dystric Brunisol | Mountain region, Old Entrance | LFH | 4.8 | 38 | 1.5 | N/A (org.hor.) | N/A (org.hor.) |
|  |  | Bf | 6.3 | 1.0 | 0.5 | Loam, 12% | Mica-illite |
