# Supplementary figures and images for: Soil humic acids degrade CWD prions and reduce infectivity
Source: PLoS Pathog. 2018 Nov 29;14(11):e1007414. doi: 10.1371/journal.ppat.1007414 (PMC6264147; doi:10.1371/journal.ppat.1007414)

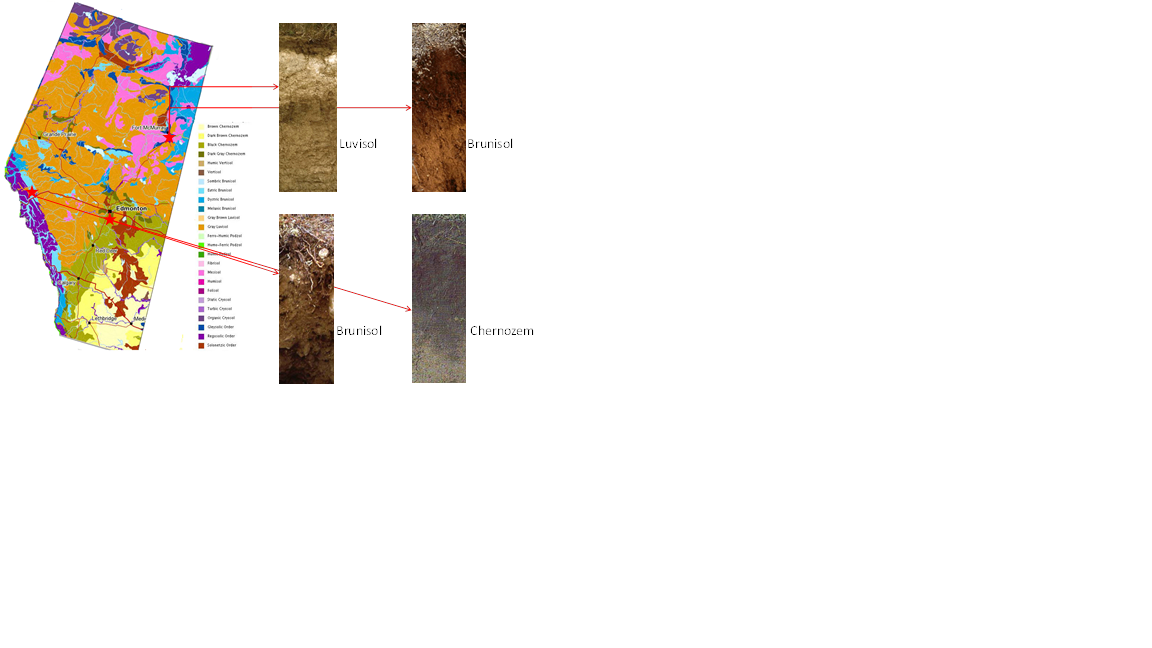

Supplement: S1 Fig — (TIF) [file ppat.1007414.s002.tif]

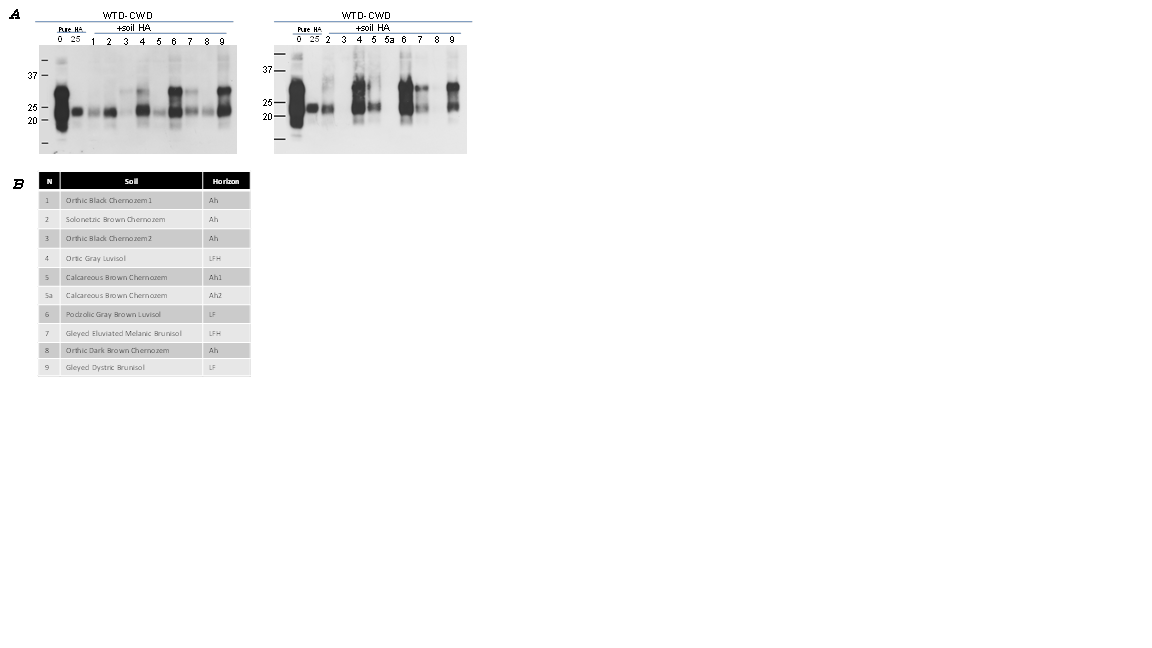

Supplement: S2 Fig — (A) Interaction of PrPCWD with pure HA and with HA extracted from different soils affects PrPCWD recovery and molecular weight. (B) Soil types used for HA extraction. Identical amounts of 10% BHCWD were incubated with water (control) and pure HA (25 g L-1 as a control) as well as HA extracted from the soils overnight at 4°C. Samples were analyzed by western blot with Bar224 antibody. (TIF) [file ppat.1007414.s003.tif]

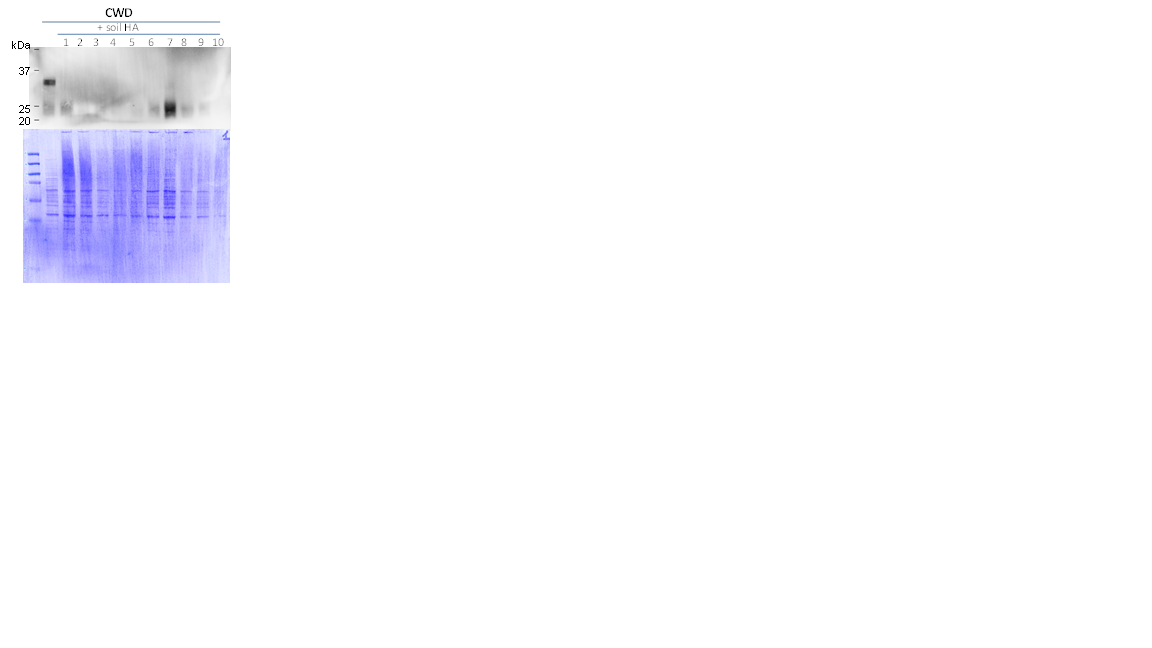

Supplement: S3 Fig — (TIF) [file ppat.1007414.s004.tif]

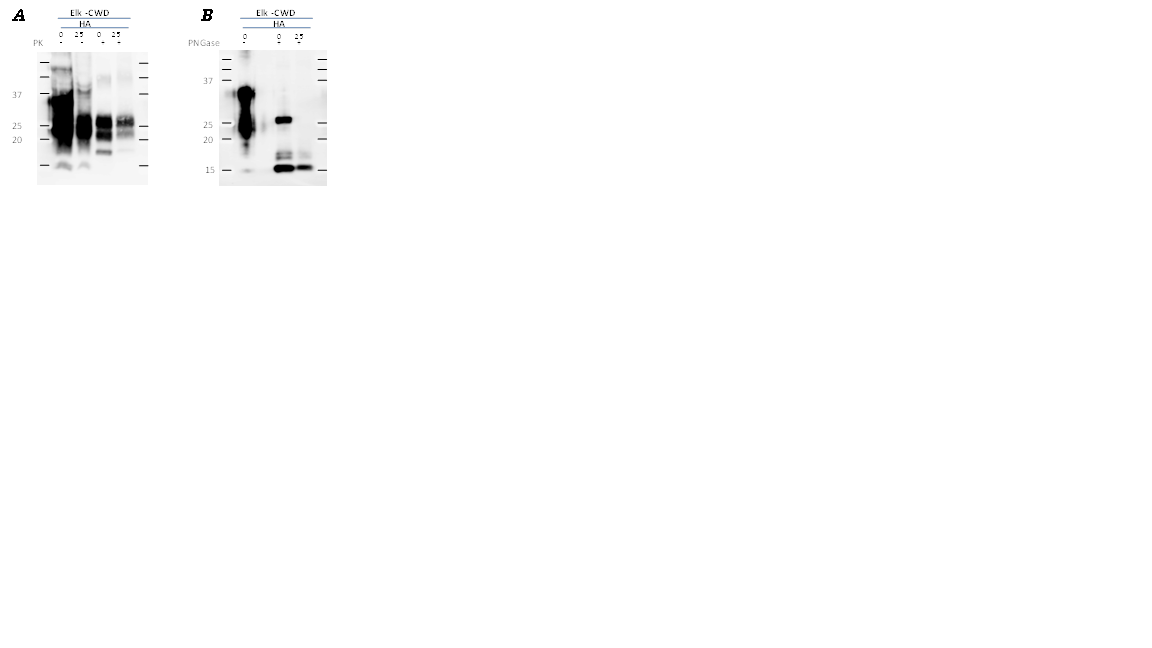

Supplement: S4 Fig — (A) High concentration of HA affects PrPres (after PK- digestion): mono- and unglycosylated forms degraded faster. (B) High concentration of HA affects deglycosylated PrPCWD (after PNGase treatment). (TIF) [file ppat.1007414.s005.tif]
